# Duration of breastfeeding is associated with leptin (*LEP*) DNA methylation profiles and BMI in 10-year-old children

**DOI:** 10.1186/s13148-019-0727-9

**Published:** 2019-08-29

**Authors:** William B. Sherwood, Victoria Bion, Gabrielle A. Lockett, Ali H. Ziyab, Nelís Soto-Ramírez, Nandini Mukherjee, Ramesh J. Kurukulaaratchy, Susan Ewart, Hongmei Zhang, S. Hasan Arshad, Wilfried Karmaus, John W. Holloway, Faisal I. Rezwan

**Affiliations:** 1 Human Development and Health, Faculty of Medicine, University Hospital Southampton, University of Southampton, Duthie Building, MP808, Tremona Road, Southampton, Hampshire, SO16 6YD UK; 2 Department of Community Medicine and Behavioral Sciences, Faculty of Medicine, Kuwait University, Kuwait City, Kuwait; 3 College of Social Work, University of South Carolina, Columbia, SC USA; 4 Division of Epidemiology, Biostatistics and Environmental Health, School of Public Health, University of Memphis, 236A Robison Hall, Memphis, TN 38152 USA; 5 Clinical and Experimental Sciences, Faculty of Medicine, University of Southampton, Southampton, UK; 6 The David Hide Asthma and Allergy Research Centre, Isle of Wight, UK; 7 College of Veterinary Medicine, Michigan State University, East Lansing, MI USA

**Keywords:** BMI trajectories, Breastfeeding, DNA methylation, Exclusive breastfeeding, Epigenetics, Obesity, *Leptin*

## Abstract

**Background:**

Breastfeeding is protective against many long-term diseases, yet the mechanisms involved are unknown. Leptin gene (*LEP*) is reported to be associated with body mass index (BMI). On the other hand, breastfeeding duration has been found to be associated with DNA methylation (DNAm) of the *LEP* gene. Therefore, epigenetic regulation of *LEP* may represent the mechanism underlying the protective effect of breastfeeding duration against obesity.

**Methods:**

In the Isle of Wight Birth Cohort, peripheral blood DNAm at 23 cytosine-phosphate-guanine sites (CpGs) in the *LEP* locus in 10-year-old (*n* = 297) samples and 16 CpGs in 18-year-old (*n* = 305) samples, were generated using the Illumina Infinium MethylationEPIC and HumanMethylation450 Beadchips respectively and tested for association with breastfeeding duration (total and exclusive) using linear regression. To explore the association between breastfeeding durations and genome-wide DNAm, epigenome-wide association studies (EWASs) and differential methylation region (DMR) analyses were performed. BMI trajectories spanning the first 18 years of life were used as the outcome to test the association with breastfeeding duration (exposure) using multi-nominal logistic regression. Mediation analysis was performed for significant CpG sites.

**Results:**

Both total and exclusive breastfeeding duration were associated with DNAm at four *LEP* CpG sites at 10 years (*P* value < 0.05), and not at 18 years. Though no association was observed between breastfeeding duration and genome-wide DNAm, DMR analyses identified five significant differentially methylated regions (Sidak adjusted *P* value < 0.05). Breastfeeding duration was also associated with the early transient overweight trajectory. Furthermore, DNAm of *LEP* was associated with this trajectory at one CpG site and early persistent obesity at another, though mediation analysis was not significant.

**Conclusions:**

Breastfeeding duration is associated with *LEP* methylation at age 10 years and BMI trajectory. *LEP* DNAm is also significantly associated with BMI trajectories throughout childhood, though sample sizes were small. However, mediation analysis did not demonstrate that DNAm of *LEP* explained the protective effect of breastfeeding against childhood obesity.

**Electronic supplementary material:**

The online version of this article (10.1186/s13148-019-0727-9) contains supplementary material, which is available to authorized users.

## Introduction

The multiple health benefits of breastfeeding are well recognised, with the World Health Organization (WHO) recommending that infants should be exclusively breastfed up until 6 months and then breastfed alongside complementary food until 2 years of age [1], though globally, a high proportion of infants are not breastfed according to international guidelines [2, 3].

Infants who are not breastfed are at increased risk of all-cause and infection-related mortality [4]. For infants with higher intensity of breastfeeding, there is a reduction in morbidities such as respiratory infection and diarrhea in their first five years of life [5]. Long-term, adequate breastfeeding has been associated with a range of phenotypes including a lowered risk of type 2 diabetes [6], improved performance in intelligence tests [7], and reduced risk of childhood leukemia [8]. Conversely, associations between breastfeeding and later disease risk are not always as clear cut, such as in the context of allergic disease [9–11]. Methodical disparities including defining exposure and adjustment for confounding factors make the comparison between studies difficult [12]. Similarly, conflicting results on the relationship between breastfeeding and obesity have been attributed to heterogeneity between studies, residual confounding, and insufficient statistical power [13]. However, several studies and meta-analyses have recently reported a significant association between breastfeeding duration and childhood obesity [6, 14, 15]. Based on current evidence, the WHO reports that breastfeeding appears to reduce the risk of being overweight and obese in childhood [16].

Early life nutrition, and breastfeeding in particular, up to 2 years of life, are considered to be critical for programming of long-term health. The composition of human breastmilk, as a source of nutrition and bioactive factors, is crucial in understanding the mechanism behind its beneficial effect [17]. For example, breastmilk is high in long-chain polyunsaturated fatty acids (LCPUFAs), which in adults are associated with a lower risk of metabolic syndrome and cardiovascular disease [18], though evidence of their benefit in children is less clear [19, 20]. Moreover, maternal antibodies in human milk are taken up by an infant’s intestinal dendritic cells to support their immature acquired immunity [17]. Exactly how these metabolites and antibodies convey the long-term protective effects of breastmilk needs further investigation [21], though recent evidence suggests that some may be directly related to epigenetic changes [22] and infants’ gut microbiota [23].

A variety of nutritional sources are thought to induce epigenetic changes, including changes in DNA methylation (DNAm) [24]. In particular, early life nutritional exposures, such as maternal protein restriction in pregnancy and methyl donor nutrients including folate, methionine, and some B-vitamins, may leave long-lasting changes to DNAm in humans [25, 26]. Furthermore, components of human breastmilk are thought to induce epigenetic changes such as to LCPUFAs [27], and lactoferrin, which by suppressing the NF-κB signaling pathway may explain why breastmilk is protective against neonatal necrotizing enterocolitis [22].

However, to date, few studies have investigated the association between breastfeeding duration and DNAm levels [28, 29]. In one of these studies, Obermann-Borst and colleagues demonstrated that breastfeeding duration was significantly associated with a reduction in methylation in whole blood DNA of the leptin gene (*LEP*) in young children (mean age 17 months) [28]. *LEP* is expressed in adipocytes and encodes the hormone leptin, important in the regulation of energy intake by inducing early satiety. However, defective production of *LEP* is associated with obesity [30], and *LEPR/LEPROT* DNAm has been demonstrated to interact with genotype to influence both leptin levels and BMI [31]. This suggests that breastfeeding duration, associated with postnatal DNAm, may contribute to childhood obesity. The aim of this study was to replicate and extend the study by Obermann-Borst et al., in the Isle of Wight Birth Cohort (IOWBC) to test the association between breastfeeding and DNAm of cytosine-phosphate-guanines (CpGs) in *LEP* gene in peripheral blood at later ages (10 and 18 years) and further explore the association between breastfeeding duration and body mass index (BMI), where DNAm may act as a mediator.

## Methods

### Isle of Wight Birth Cohort

The IOWBC (second generation also known as IoW F_1_) was recruited between 1989 and 1990 (*n* = 1536) [32, 33]. The parents (first generation, IoW F_0_) of all infants born over this period were contacted at birth, and subsequently, 95% of infants (*n* = 1456) were enrolled following informed consent and exclusion. Follow-ups were conducted at 1, 2, 4, 10, and 18 years, where information was gathered about the participants, such as infant nutrition and breastfeeding practice [34]. Peripheral blood samples were collected from participants at age 10 and 18 years and DNAm measured for 297 and 305 samples, at ages 10 and 18 respectively, of whom 162 participants were matched between the two groups. Descriptive statistics of the sub-cohorts for exposure and covariates are presented in Table 1.

**Table 1:** Characteristics of Isle of Wight Birth Cohort participants at 10 and 18 years.

Characteristic	IOWBC Participants at 10 years (*n* = 297)	IOWBC Participants at 18 years (*n* = 305)
Child sex (female)	190 (64.0%)	209 (68.5%)
Maternal socioeconomic status		
1 (lowest)	41 (13.8%)	49 (16.1%)
2	60 (20.2%)	52 (17.0%)
3	89 (29.9%)	88 (28.9%)
4	82 (27.6%)	85 (27.9%)
5 (highest)	25 (8.4%)	31 (10.2%)
No maternal smoking during pregnancy	238 (80.1%)	250 (82.0%)
Gestational age < 38 weeks	12 (4.0%)	15 (4.9%)
Overweight (BMI > 19.8 at 10 years, > 25 at 18)	62 (20.9%)	83 (27.2%)
Never breastfed	38 (12.8%)	48 (15.7%)
BMI trajectory		
Normal (reference)	211 (71%)	215 (70.5%)
Delayed overweight (trajectory 2)	41 (13.8%)	48 (15.8%)
Early transient overweight (trajectory 3)	33 (11.1%)	30 (9.8%)
Early persistent obesity (trajectory 4)	12 (4.1%)	12 (3.9%)

Descriptive statistics on exposure and covariates of participants included in the study. This is given as the number of participants in each category and as a percentage for each characteristic. Note that for the overweight category, different BMI cut-offs are used at 10 or 18 years, as defined by the International Obesity Taskforce

### Categorization of breastfeeding duration

Two categories, exclusive and total breastfeeding durations, were used to define the duration of breastfeeding for each participant. The exclusive breastfeeding duration was defined as the length of time (in weeks) a child was breastfed, until formula feed and/or solid foods were introduced. The total breastfeeding duration, equates to the total number of weeks a mother breastfed her child irrespective of the introduction of formula feed and/or solid foods.

### DNA extraction and arraying

DNA was extracted from peripheral blood samples using a standard salting out procedure. Approximately 1 μg of DNA was bisulfite-treated using the EZ 96-DNA methylation kit (Zymo Research, Irvine, CA, USA) using the manufacturer’s standard protocol. The Infinium MethylationEPIC BeadChips (age 10-year samples) and Infinium HumanMethylation450 (age 18-year samples) from Illumina (Illumina, San Diego, CA, USA) were used to obtain methylation levels following the manufacturer’s standard protocol. Methylation 450k and EPIC data (β values) were pre-processed for quality control, following the CPACOR pipeline [35], and batch effect was corrected using ComBat [36].

### Candidate *Leptin* CpG selection

In Obermann-Borst et al. study, seven CpG sites in the *LEP* gene were analyzed. The UCSC Batch Coordinate Conversion tool (LiftOver, https://genome.ucsc.edu/cgi-bin/hgLiftOver) was used to convert genome coordinates from the hg18 assembly, used by Obermann-Borst et al., to the human genome hg19 assembly [37]. Four of these seven CpG sites were present in our analysis. For all CpGs annotated near *LEP* gene, for the 18-year samples, a total of 16 CpG sites were analyzed in *LEP*, whereas 23 CpG sites in *LEP* were analyzed in the 10-year samples, where the more comprehensive EPIC beadchips were used (see Additional file 1: Table S1).

### Confounding factors

The same covariates used by Obermann-Borst et al. were used in the analysis, namely, child sex, birth weight, gestational age, serum leptin (ng/mL at either 10 or 18 years), BMI (at either 10 or 18 years), maternal socioeconomic status (defined using maternal socioeconomic cluster information: high, low, low-low, low-mid, and mid, using household income, number of rooms, and maternal education), and maternal smoking at birth. However, unlike the original study, birth weight, and gestational age were kept as separate variables and *z*-scores were not considered to be appropriate [38]. Parity and maternal age were added as covariates for these analyses, having been previously found to be associated with breastfeeding duration [39]. Additionally, breastfeeding duration was used as a continuous variable rather than categorical. Cell proportions (CD8T, CD4T, NK, B Cells, monocytes, granulocytes) were estimated using the minfi package [40] and cell composition coefficients were derived using the Houseman method [41].

### BMI trajectories

Four BMI trajectories (normal, early persistent obesity, delayed overweight, and early transient overweight) covering the period from 1 to 18 years of age, as proposed by Ziyab et al. [42], were utilized to assess the association between breastfeeding duration and BMI trajectories. These four trajectories were categorized using age-specific BMI thresholds for ‘obesity’ and ‘overweight,’ as defined by the International Obesity Taskforce for ages 4, 10 and 18 years [43], and WHO standards for age 1 year [44]. They were defined in 1240 participants of the IOWBC and the trajectories for 10 and 18-year samples are described in Table 1.

### Statistical analyses

Intra-class correlations (ICC) were estimated among 14 common *LEP* CpGs in 162 matched samples (common between 10 and 18 years) to assess the DNAm stability over time. Four models were run exploring the association between exclusive breastfeeding duration or total breastfeeding duration and *LEP* DNAm at either 10 or 18 years using linear regression adjusted for covariates and cell types.

Further, we performed an epigenome-wide association study (EWAS) to identify differentially methylated positions (DMPs) that are associated with breastfeeding durations (both exclusive and total), using linear regression adjusted for confounding factors and cell types, in both 10- and 18-year samples as described for the *LEP* analyses. Multiple hypothesis testing was accounted for by controlling the false discovery rate (FDR), using Benjamini and Hochberg’s algorithm [45]. CpGs with FDR corrected *P* value < 0.05 were considered statistically significant. To identify differentially methylated regions (DMRs), composed of multiple signals across individual CpG positions, comb-p [46] (Python v2.7) was used. Comb-p identifies regions enriched with low unadjusted *P* values (here, *P* value < 0.05) from the EWAS analysis, based on the probe location. For each region, the comb-p algorithm adjusts the CpG *P* values for auto-correction between probes by using the Stouffer-Liptak-Kechris (slk) correction with multiple testing adjustment using a one-step Sidak correction method. Regions with least two CpG probes within 200 base pairs, having a Sidak-corrected *P* value < 0.05, were considered statistically significant.

The association between breastfeeding duration (exclusive and total) and BMI trajectories was tested in the subset of children, matched with 10-year old samples, using multi-nominal logistic regression with ‘normal’ BMI trajectory as reference. Furthermore, the association between BMI trajectory and *LEP* DNAm was investigated with only those CpGs found to be significant with breastfeeding duration. *P* value < 0.05 was used as the significance level. All analyses were performed using R (version 3.3.2).

### Mediation analysis

**Fig. 1:**
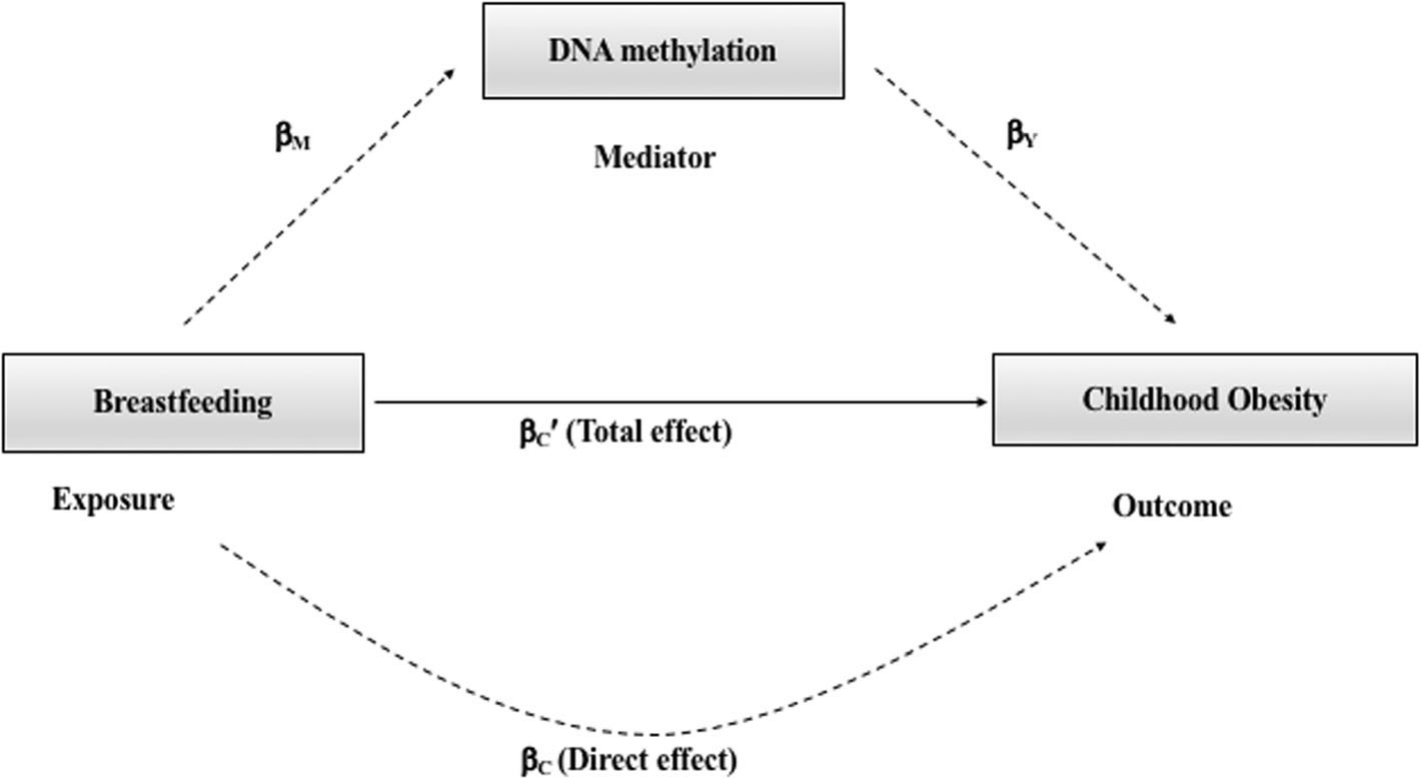
A mediation model for the association between breastfeeding duration (total and exclusive) and childhood obesity. β_M_ represents the effect estimate for breastfeeding duration on DNA methylation (CpG ~ breastfeeding duration + covariates); β_Y_ represents the effect estimate for CpG on childhood obesity (BMI trajectory ~ CpG + covariates; βc represents the direct effect (no mediation) estimate for breastfeeding duration on childhood obesity (BMI trajectory ~ breastfeeding duration + covariates; βc′ represents the total effect estimate on childhood obesity (BMI trajectory = breastfeeding duration + covariates + CpG)

Model-based causal mediation analysis, using the R-package “mediation” [47], was performed to explore whether the association between breastfeeding duration and BMI trajectory is mediated by DNAm (Fig. 1). Only the *LEP* CpGs significantly associated with BMI trajectory were utilized for the mediation analysis as the mediator. Firstly, we estimated the effect of breastfeeding duration (exposure) on *LEP* DNAm (mediator) and then, we estimated the combined effect of breastfeeding duration (exposure) and *LEP* DNAm (mediator) on the BMI trajectory (outcome). Both of these effects were adjusted for the covariates mentioned above. Bootstrapping was used to test significance level (*P* value < 0.05).

## Results

### Stability of DNA methylation

Intra-class correlation shows that 64.3% of the common CpGs (9 out of 14) were fairly moderately stable (ICC between 0.05 to 0.75) over 8 years (Additional file 1: Table S2). A moderate-good degree of stability was found in three CpGs between the two measurements. The highest ICC observed (in cg19594666) was 0.69 with a 95% confidence interval from 0.58 to 0.77 (*F*(161,161) = 3.22, *P* value = 2.79 × 10^−13^).

### Breastfeeding duration and *LEP* DNA methylation

At 10 years, the total breastfeeding duration was significantly associated with DNAm at four CpG sites in *LEP* (*P* value < 0.05, Table 2). Two of the CpGs (cg03084214 and cg23753947) were available in 18-year samples, which showed significant differences with 10-year samples (Additional file 1: Figure S1). However, for the 18-year samples, there were no significant associations seen between either total breastfeeding or exclusive breastfeeding duration and DNAm at any of the 16 *LEP* CpG sites interrogated (Additional file 1: Tables S3 and S4).

**Table 2:** Association between total breastfeeding duration and *LEP* DNAm at 10 years

Probe name	Estimate	SE	*P* value
cg03084214	5.23E−04	2.33E−04	0.026
cg11005360	− 2.39E−04	1.07E−04	0.027
cg23381058	− 4.91E−04	1.96E−04	0.013
cg23753947	1.73E−04	8.20E−05	0.036

Columns: *Probe name* = *LEP* CpG sites from Illumina Infinium MethylationEPIC Beadchip significantly associated with total breastfeeding duration; *Estimate* = coefficient denoting the increase or decrease in methylation by one unit (in week) of increase of total breastfeeding duration; *SE* = standard error of coefficient; *P* value < 0.05 was considered significant. Only the four CpG sites considered significant are included, see Additional file 1: Table S5, for all 23 CpG sites tested

Similarly, the duration that a child was exclusively breastfed was associated with significant changes (*P* value < 0.05) in DNAm at four CpG sites, in samples taken at 10 years (Table 3). Lower methylation of one site (cg23381058) was significantly associated with both exclusive and total breastfeeding duration. Another CpG (cg18603538) was available in 18-year samples, which showed no significant differences with 10-year samples (Additional file 1: Figure S1).

**Table 3:** Association between exclusive breastfeeding duration and *LEP* DNAm at 10 years

Probe name	Estimate	SE	*P* value
cg05091920	4.23E−04	2.12E−04	0.048
cg15792829	5.87E−04	2.37E−04	0.015
cg18603538	8.07E−04	3.69E−04	0.030
cg23381058	− 8.17E−04	4.00E−04	0.043

Columns: *Probe name* = *LEP* CpG sites from Illumina Infinium MethylationEPIC Beadchip significantly associated with exclusive breastfeeding duration; *Estimate* = coefficient denoting the increase or decrease in methylation by one unit (in week) of increase of exclusive breastfeeding duration; *SE* = standard error of coefficient; *P* value < 0.05 was considered significant. Only the four CpG sites considered significant are included, see Additional file 1: Table S6, for all 23 CpG sites tested.

### Genome-wide DMPs and DMRs identifications

The EWAS analyses for both exclusive and total breastfeeding did not identify any significant DMPs (FDR < 0.05) at either age 10 or 18 years (Additional file 1: Figures 2–5). Analysis of differentially methylated regions using comb-p identified five significant DMRs (Sidak-corrected *P* values 9.6 × 10^−9^−0.005) (Tables 4 and 5). We identified two DMRs for 10-year samples and one for 18-year samples in the case of total breastfeeding duration. For exclusive breastfeeding duration, we identified two significant DMRs for 18-year samples and none for the 10-year samples. There was no overlap between the DMRs identified for exclusive and total breastfeeding.

**Table 4:** Statistically significant differentially methylated regions (DMRs) (Sidak *P* value < 0.05) for total breastfeeding duration

Age	Location	No. of probes	Slk *P* value	Sidak *P* value	Ref gene name and genomic feature		CpG feature
10	chr10:104535854-104535920	2	4.62E−07	0.005	WBP1L	TSS200; body; 5′UTR;1stExon	NA
10	chr19:54567060-54567128	3	3.74E−07	0.004	VSTM1	5′UTR;1stExon; body	NA
18	chr22:37678728-37678791	2	2.49E−07	0.002	CYTH4	Body	NA

*Both Slk* = uncorrected Stouffer-Liptak-Kechris *P* values, and Sidak *P* values corrected for multiple testing are reported. Here, 5′UTR = 5 prime untranslated region; *TSS200* = 0–200 bases upstream from the transcription start sites; *TSS1500* = 200–1500 bases upstream from the transcriptional start site; *body* = gene body

**Table 5:** Statistically significant differentially methylated regions (DMRs) (Sidak *P* value < 0.05) for exclusive breastfeeding duration

Age	Location	No. of probes	Slk *P* value	Sidak *P* value	Ref gene name and genomic feature		CpG feature
18	chr1:173837002-173837197	7	4.13E−12	9.62E−09	GAS5	Body; TSS150;TSS200	Island, shore
18	chr2:24397787-24397845	3	1.43E−07	0.001	FAM228A	TSS200	Island

*Both Slk* = uncorrected Stouffer-Liptak-Kechris *P* values, and Sidak *P* values corrected for multiple testing are reported. Here, 5′UTR = 5 prime untranslated region; *TSS200* = 0–200 bases upstream from the transcription start sites; *TSS1500* = 200–1500 bases upstream from the transcriptional start site; *body* = gene body

### Breastfeeding duration and BMI trajectories

Total duration of breastfeeding was significantly associated with the early transient overweight BMI trajectory (estimate = − 0.02, *P* value = 0.002) (Table 6). However, there was no association with the early persistent obesity or delayed overweight trajectories. A similar pattern was seen when testing exclusive breastfeeding duration and obesity (Table 6), though the association between exclusive breastfeeding duration and early transient overweight narrowly missed our threshold for significance (*estimate* = − 0.03, *P* value = 0.05).

**Table 6:** Association between duration of total and exclusive breastfeeding and BMI trajectory

	Total breastfeeding				Exclusive breastfeeding		
BMI trajectory	Estimate	SE	*P* value		Estimate	SE	*P* value
2	0.007	0.007	0.29		0.002	0.014	0.88
3	− 0.02	0.008	0.002*		− 0.03	0.015	0.05
4	0.012	0.014	0.40		− 0.02	0.030	0.43

BMI trajectory defined by Ziyab et al. [42]. Columns: *trajectory 2* = early persistent obesity, *trajectory 3* = early transient overweight, *trajectory 4* = delayed overweight; *estimate* = coefficient denoting one-unit (in week) increase in breastfeeding duration is associated with the increase in the log odds of being in BMI trajectory; *standard error* = standard error of coefficient. *P* value < 0.05 was considered significant and is indicated by *. Each trajectory was tested against the control group (BMI trajectory 1)

### DNA methylation and BMI Trajectories

Of the four CpG sites where total breastfeeding duration was associated with DNAm, one site (cg23381058) also had a significant association between DNAm and the early transient overweight trajectory (*P* value = 0.034) (Table 7). Note that this CpG site was associated with both total and exclusive breastfeeding duration. Of the four sites where exclusive breastfeeding was associated with DNAm, methylation at another site (cg05091920) was significantly associated with the early persistent obesity trajectory (*P* value = 0.028). These results have been placed in Additional file 1: Table S7 along with other non-significant CpGs for all of the trajectories.

**Table 7:** Mediation analysis between cg23381058, total breastfeeding duration and BMI Trajectory 3 (Early transient overweight)

Total breastfeeding				Exclusive breastfeeding			
Probe name	Estimate	SE	*P* value	Probe name	Estimate	SE	*P* value
cg03084214	6.585	7.330	0.349	cg05091920	− 15.843	10.651	0.125
cg11005360	2.986	15.504	0.366	cg15792829	− 17.064	9.602	0.155
cg23381058	3.183	4.277	0.034*	cg18603538	− 1.101	6.033	0.292
cg23753947	-23.567	17.710	0.946	cg23381058	4.805	3.187	0.034*

Table 7 Association between early transient overweight (BMI trajectory 3) and *LEP* DNAm at 10 years previously associated with breastfeeding duration (associated *P* values are shown)

Columns: *Probe name* = *LEP* CpG sites from Illumina Infinium MethylationEPIC Beadchip, which are significantly associated with either total or exclusive breastfeeding; *Estimate* = coefficient denoting one-unit increase in the methylation level for specific CpG is associated with the decrease in the log odds of being in the BMI trajectory 3; *standard error* = standard error of coefficient. DNA methylation analyzed against BMI trajectories with BMI trajectory 1 (normal) as a control. *P* value < 0.05 was considered significant and indicated by *

### Mediation analysis

There were no statistically significant effects of cg23381058 methylation as a mediator between total breastfeeding duration and the early transient overweight trajectory (Table 8). Similarly, there was no association when looking at exclusive breastfeeding duration (Table 8). In the case of total breastfeeding duration, no significant direct effect (*P* value = 0.06) or total effect (*P* value = 0.07) on early transient overweight trajectory was observed.

**Table 8:** Mediation analysis between cg23381058, total breastfeeding duration, and BMI trajectory 3 (early transient overweight)

	Total breastfeeding duration				Exclusive breastfeeding duration			
	Beta	95% CI Lower	95% CI Upper	*P* value	Beta	95% CI Lower	95% CI Upper	*P* value
ACME	5.81E−05	− 2.46E−04	4.31E−04	0.73	2.48E−04	− 3.1E−04	1.19E−03	0.42
ADE	− 2.43E−03	− 4.94E−03	1.16E−04	0.06	− 2.25E−03	− 7.27E−03	2.76E−03	0.36
Total effect	− 2.37E−03	− 4.91E−03	1.42E−04	0.07	− 2.00E-03	− 7.07E−03	3.06E−03	0.42
Prop. mediated	− 2.45E−02	− 4.19E−01	2.72E−01	0.76	− 0.12	− 1.66	1.54	0.72

Columns: Effects— *ACME* average causal mediation effects, *ADE* average direct effects, *Total effect*, sum of a mediation (indirect) effect and a direct effect, *Prop. mediated* proportion of the total effect explained by the mediator

## Discussion

We examined the association between breastfeeding duration and DNAm on the *LEP* gene, finding a significant association at 10 years but not at 18 years. Exclusive breastfeeding duration was significantly associated with DNAm at four CpGs and total breastfeeding was also associated with DNAm at four sites, and among them, one site, cg23381058, was in common. Previous studies investigating the association between breastfeeding and DNAm, in animal and human models, have used total breastfeeding duration as their exposure [48]. However, there are concerns about the variability of breastfeeding within these parameters. For example, infants could be largely formula-fed, yet still classified as breastfeeding. Using an exclusive breastfeeding category, in comparison with total breastfeeding, gives more confidence over the exposure variable. The importance of this distinction is highlighted by ongoing research into impact of breastfeeding on infant outcomes, shaping health policy [1].

In addition, epigenome-wide association analysis was undertaken to identify other DMPs and DMRs associated with the breastfeeding duration (exclusive and total) and for both 10- and 18-year samples. To our knowledge, this is the first time such an analysis, investigating the association between breastfeeding and DNAm, has been conducted using an EWAS approach. In the case of DMPs, no genome-wide significant (FDR < 0.05) hits were found. This is likely due to lack of power and follow up studies in larger cohorts and/or with DNAm measurements earlier in childhood are warranted. We did however identify five significant DMRs (Sidak-corrected *P* values < 0.05) associated with breastfeeding duration. One of these genes, *GAS5*, was reported in a recent paper exploring whether long non-coding RNAs (lncRNAs), important in the development and gene expression, were encapsulated in extracellular vesicles within breastmilk [49]. Among others, *GAS5* was detected and indeed highly correlated. Involved in metabolic functions and immune system programming, and as a significant epigenetic regulator, *GAS5* is a potential candidate breast-milk compound, that could be important in the effect of breastfeeding. We are not aware of any previous research linking breastfeeding with the remaining DMRs; *WBP1L*, *CYTH4*, *VSTM1*, and *FAM228A*. Interestingly though, a recent study by Rzehak et al. [50], found an association between DNAm at cg14518658 near *CYTH4* gene and body composition in pre-school children, adjacent to one of the significant DMRs (chr22:37678728-37678791) identified in this study.

The study by Obermann–Borst et al. demonstrated that breastfeeding is associated with DNAm in *LEP* gene at 17 months of age. Obermann-Borst et al. hypothesized that induced epigenetic regulation of leptin expression may be mechanistic in the protective effect of breastfeeding against obesity (Fig. 1). Our results support Obermann-Borst et al. findings, at a later stage in childhood (10 years) but not into young adulthood (18 years). However, though significant, these effect sizes were small and we saw varying direction of effect between probes. Stability of DNAm at cg23381058 over time could not be assessed as it was not present on both DNAm Bead Chip platforms. However, the majority of CpGs exhibited moderate stability over time. This change in DNAm may be a response to other environmental stimuli by 18 years, and indeed it is recognized that DNAm levels in an individual alter throughout childhood [51].

The next stage was to examine whether breastfeeding duration is associated with obesity in the IOWBC. The BMI trajectories, devised by Ziyab et al. [42], incorporate the different age-specific (BMI) thresholds of obesity proposed by the International Obesity Task Force and WHO, allowing analysis of the maintenance of this association throughout childhood. A significant association between total breastfeeding duration and the ‘early transient overweight’ BMI trajectory was seen in the subset of children, matched to 10-year-old samples. It should be noted that the sample size in this trajectory was considerably smaller (*n* = 33 at age 10) than in the normal reference category (*n* = 211). No association was seen between total breastfeeding duration and early persistent obesity or delayed overweight trajectory. Again the sample sizes in these trajectories were far smaller (*n* = 12 at age 10 in early persistent obesity group) than the reference trajectory; therefore, a significant effect of breastfeeding may not have been seen due to low power. Nonetheless, these results suggest breastfeeding duration may be involved in a short-term inverse association with childhood obesity. Another independent study examining the BMI trajectories of 276 infants, found that formula-fed infants gain weight more rapidly and disproportionately than breastfed infants [52]. This was attributed to increased lean mass, suggesting that delayed obesity associated with breastfeeding is not related to increased adiposity in infancy. This evidence contradicts our results, which indicates that breastfeeding is independently associated with both DNAm and obesity in childhood, but not long-term.

Multiple studies have demonstrated that DNAm at a number of loci is significantly associated with childhood obesity, for example in *HIF3A* gene [53], as well as leptin and *adiponectin*, where DNAm is also associated with low-density lipoprotein cholesterol levels [54, 55]. However, in this case, *LEP* DNAm levels have been reported to be negatively associated with BMI, contrasting with the hypothesis by Obermann-Borst et al., who suggested that hypo-methylation on the *LEP* gene may protect against obesity, i.e., reduce BMI. Indeed, we found a mixture of increased and decreased methylation indicating the relationship may not be simple. We also sought to test the association between DNAm and BMI trajectory in the IOWBC. DNAm at the CpG site cg23381058, which had shown a significant negative association with total and exclusive breastfeeding duration, was found to be negatively associated with early transient overweight, though mediation analysis of this CpG site was not significant (*P* < 0.05). This CpG site is located within an intron of *LEP*, in a transcription factor binding site. Conversely, an effect was seen at cg05091920, though here a decrease in the methylation level was associated with the decrease in the log odds of being in early persistent obesity trajectory.

We recognize that there are some limitations to this study. Firstly, we had no measurements of DNAm in infancy and thus our mediation analysis is based on the assumption that DNAm at age 10 years associated with breastfeeding duration is stable from infancy. Most importantly, it is difficult to establish a causal relationship between breastfeeding, DNAm, and obesity. It has been previously reported that changes in DNAm are a consequence of obesity [56–58]; however, these studies were largely conducted in adult populations, and inter-study comparison is difficult. Furthermore, breastfeeding may alter DNAm in a tissue-specific manner, and what effect this DNAm has on *LEP* expression as well as tissue specificity must be explored. Whilst this study and that of Obermann-Borst et al. used peripheral blood samples, and leptin hormone circulates through the blood, the *LEP* gene is known to be expressed in adipocytes, questioning the biological relevance. However, it has been demonstrated that epigenetic markers in blood accurately reflect those in adipose tissue [54, 55]. Peripheral blood remains an effective tissue from which to measure DNAm in childhood, though the investigation of alternative tissues is of interest.

The findings of this study require replication in other cohorts with childhood data and larger sample sizes to improve our understanding of how the relationship between breastfeeding, DNAm, and obesity varies throughout childhood. Cohorts with methylation data from cord blood and matched participants later in childhood could also help to establish causality. Epigenome-wide association studies have helped identify thousands of differentially methylated genes, associated with disease processes and/or environmental processes. This will be an important step to identify the importance of DNAm as a mechanism propagating the long-term protective effects of breastfeeding.

## Conclusion

In line with previous studies, a significant association of breastfeeding duration with altered methylation at seven CpG sites on *LEP* gene was observed, and an association of breastfeeding duration with childhood obesity was confirmed. Future studies in larger population samples are warranted to further explore the potential of DNAm at *LEP* and other sites in the genome in mediating the effect of breastfeeding on childhood obesity.

## Additional file

Additional file 1: **Table S1.** Annotation of *LEP* CpGs used in the analyses. **Table S2.** Stability of *LEP* CpGs (between 10 and 18 years).**Table S3.** Association between total breastfeeding duration and *LEP* DNAm (16 CpGs) at 18 years. **Table S4.** Association between exclusive breastfeeding duration and *LEP* DNAm (16 CpGs) at 18 years. **Table S5.** Association between total breastfeeding duration and *LEP* DNAm (23 CpGs) at 10 years. **Table S6.** Association between exclusive breastfeeding duration and *LEP* DNAm (23 CpGs) at 10 years. **Table S7.** Association between BMI trajectories and *LEP* DNAm (at 10 years) at CpGs previously associated with total or exclusive breastfeeding duration (associated P-values have been shown). **Figure S1.** Comparisons of methylation level (beta) of significant common CpGs in matched 162 samples (10 and 8 years). (DOCX 3070 kb)

## Data Availability

The datasets used and/or analyzed during the current study are available from the corresponding author on reasonable request. For access to the full Isle of Wight Cohort data please see: http://www.allergyresearch.org.uk/studies/birth-cohort/-cohort-data-use

## Abbreviations

CpG: Cytosine-phosphate-guanine
DNAm: DNA methylation
IOWBC: Isle of Wight Birth Cohort
LCPUFAs: Long-chain polyunsaturated fatty acids
*LEP*: *Leptin*
WHO: World Health Organization
