# Supplementary material for: Duration of breastfeeding is associated with leptin (LEP) DNA methylation profiles and BMI in 10-year-old children
Source: Clin Epigenetics. 2019 Aug 29;11:128. doi: 10.1186/s13148-019-0727-9 (PMC6716837; doi:10.1186/s13148-019-0727-9)
Supplement: Supplementary file 1 — Table S1. Annotation of LEP CpGs used in the analyses. Table S2. Stability of LEP CpGs (between 10 and 18 years).Table S3. Association between total breastfeeding duration and LEP DNAm (16 CpGs) at 18 years. Table S4. Association between exclusive breastfeeding duration and LEP DNAm (16 CpGs) at 18 years. Table S5. Association between total breastfeeding duration and LEP DNAm (23 CpGs) at 10 years. Table S6. Association between exclusive breastfeeding duration and LEP DNAm (23 CpGs) at 10 years. Table S7. Association between BMI trajectories and LEP DNAm (at 10 years) at CpGs previously associated with total or exclusive breastfeeding duration (associated P-values have been shown). Figure S1. Comparisons of methylation level (beta) of significant common CpGs in matched 162 samples (10 and 8 years). (DOCX 3070 kb) [file 13148_2019_727_MOESM1_ESM.docx]

**Additional file 1**

**Table S1:** Annotation of *LEP* CpGs used in the analyses.

| Probe name | Chr | Mapinfo | UCSC CpG Island Name | Relation to UCSC CpG Island | Age group |
| --- | --- | --- | --- | --- | --- |
| cg00666422 | 7 | 127881440 | chr7:127880750-127881375 | S_Shore | 10Y,18Y |
| cg00840332 | 7 | 127881269 | chr7:127880750-127881375 | Island | 10Y,18Y |
| cg03084214 | 7 | 127880619 | chr7:127880750-127881375 | N_Shore | 10Y,18Y |
| cg04833007 | 7 | 127894849 | - | - | 10Y |
| cg05091920 | 7 | 127897975 | - | - | 10Y |
| cg05136031 | 7 | 127880567 | chr7:127880750-127881375 | N_Shore | 10Y |
| cg07464571 | 7 | 127881001 | chr7:127880750-127881375 | Island | 18Y |
| cg08349670 | 7 | 127882889 | chr7:127880750-127881375 | S_Shore | 10Y |
| cg11005360 | 7 | 127885181 | chr7:127880750-127881375 | S_Shelf | 10Y |
| cg11045943 | 7 | 127881293 | chr7:127880750-127881375 | Island | 10Y |
| cg12083122 | 7 | 127883819 | chr7:127880750-127881375 | S_Shelf | 10Y,18Y |
| cg12782180 | 7 | 127880932 | chr7:127880750-127881375 | Island | 10Y,18Y |
| cg13381984 | 7 | 127881344 | chr7:127880750-127881375 | Island | 10Y,18Y |
| cg14734794 | 7 | 127879920 | chr7:127880750-127881375 | N_Shore | 18Y |
| cg15792829 | 7 | 127881747 | chr7:127880750-127881375 | S_Shore | 10Y |
| cg16683741 | 7 | 127891959 | - | - | 10Y,18Y |
| cg18603538 | 7 | 127894591 | - | - | 10Y,18Y |
| cg19594666 | 7 | 127881280 | chr7:127880750-127881375 | Island | 10Y,18Y |
| cg20564991 | 7 | 127882514 | chr7:127880750-127881375 | S_Shore | 10Y |
| cg23381058 | 7 | 127888052 | - | - | 10Y |
| cg23753947 | 7 | 127889701 | - | - | 10Y,18Y |
| cg24862443 | 7 | 127896859 | - | - | 10Y,18Y |
| cg25435800 | 7 | 127890193 | - | - | 10Y,18Y |
| cg25730670 | 7 | 127891366 | - | - | 10Y,18Y |
| cg26814075 | 7 | 127881298 | chr7:127880750-127881375 | Island | 10Y,18Y |

Columns: Probe name = Unique identifier from the Illumina CG database; Chr = Chromosome; Mapinfo = chromosomal coordinates of the CpG (hg19 build); UCSC CpG Island Name= Chromosomal coordinates of the CpG Island from UCSC; Relation to UCSC CpG Island = The location of the CpG relative to the CpG island (Shore = 0–2 kb from island; Shelf = 2–4 kb from island; N = upstream (5’) of CpG island; S = downstream (3’) of CpG island; Age group = Age group from IOWBC (10 years (10Y) or 18 years (18Y)).

**(Source:** https://support.illumina.com/content/dam/illumina-support/documents/downloads/productfiles/methylationepic/infinium-methylationepic-manifest-column-headings.pdf).

**Table S2:** Stability of *LEP* CpGs (between 10 and 18 years).

|  |  | 95% Confidence Interval | |  | F Test with True Value 0 | | | |
| --- | --- | --- | --- | --- | --- | --- | --- | --- |
| Probe name | ICC | Lower Bound | Upper Bound |  | Values | df1 | df2 | P-value |
| cg00666422 | 0.65 | 0.52 | 0.74 |  | 2.87 | 161 | 161 | 3.36E-11* |
| cg00840332 | 0.66 | 0.53 | 0.75 |  | 2.91 | 161 | 161 | 1.78E-11* |
| cg03084214 | 0.26 | 0.00 | 0.46 |  | 1.35 | 161 | 161 | 0.03* |
| cg12083122 | 0.00 | 0.00 | 0.05 |  | 0.77 | 161 | 161 | 0.95 |
| cg12782180 | 0.60 | 0.45 | 0.70 |  | 2.49 | 161 | 161 | 7.00E-09* |
| cg13381984 | 0.64 | 0.50 | 0.73 |  | 2.75 | 161 | 161 | 1.77E-10* |
| cg16683741 | 0.00 | 0.00 | 0.17 |  | 0.89 | 161 | 161 | 0.77 |
| cg18603538 | 0.39 | 0.17 | 0.56 |  | 1.65 | 161 | 161 | 8.17E-04* |
| cg19594666 | 0.69 | 0.58 | 0.77 |  | 3.22 | 161 | 161 | 2.79E-13* |
| cg23753947 | 0.46 | 0.26 | 0.60 |  | 1.85 | 161 | 161 | 5.43E-05* |
| cg24862443 | 0.00 | 0.00 | 0.22 |  | 0.93 | 161 | 161 | 0.67 |
| cg25435800 | 0.19 | 0.00 | 0.40 |  | 1.23 | 161 | 161 | 0.09 |
| cg25730670 | 0.00 | 0.00 | 0.04 |  | 0.76 | 161 | 161 | 0.95 |
| cg26814075 | 0.61 | 0.47 | 0.72 |  | 2.60 | 161 | 161 | 1.46E-09* |

Single score Intraclass Correlation (ICC) was measured between 14 *LEP* CpGs in 162 matched samples at 10 and 18 years. Here, two-way analysis of variance has been done for the model where “consistency” between the two-time point has been assessed. Columns: Probe name = Common *LEP* CpG sites in Illumina Infinium HumanMethylation450 and MethylationEPIC Beadchips; ICC = intraclass correlation coefficient; Lower Bound and Upper Bound = lower and upper bound of the coefficient with 95% confidence interval; Values = F-test values; df1 and df2 = degree of freedom 1 and 2 for the F-test; P-value = significance level of the test.

**Table S3:** Association between total breastfeeding duration and *LEP* DNAm (16 CpGs) at 18 years.

| Probe name | Beta | SE | P-value |
| --- | --- | --- | --- |
| cg00666422 | -0.0022 | 0.0018 | 0.23 |
| cg03084214 | -0.0006 | 0.0010 | 0.55 |
| cg12083122 | -0.0003 | 0.0006 | 0.61 |
| cg14734794 | 0.0001 | 0.0005 | 0.85 |
| cg16683741 | -0.0005 | 0.0006 | 0.40 |
| cg18603538 | -0.0011 | 0.0011 | 0.31 |
| cg23753947 | -0.0001 | 0.0005 | 0.83 |
| cg24862443 | 0.0001 | 0.0005 | 0.84 |
| cg25435800 | 0.0009 | 0.0009 | 0.27 |
| cg25730670 | -0.0001 | 0.0005 | 0.89 |
| cg07464571 | -0.0012 | 0.0011 | 0.29 |
| cg12782180 | -0.0012 | 0.0017 | 0.47 |
| cg13381984 | 0.0001 | 0.0016 | 0.93 |
| cg19594666 | -0.0014 | 0.0019 | 0.47 |
| cg26814075 | -0.0003 | 0.0014 | 0.83 |
| cg00840332 | -0.0015 | 0.0016 | 0.35 |

Columns: Probe name = *LEP* CpG sites from Illumina Infinium HumanMethylation450 Beadchip; Beta = coefficient from regression model; SE = standard error of coefficient.

**Table S4:** Association between exclusive breastfeeding duration and *LEP* DNAm (16 CpGs) at 18 years.

| Probe name | Beta | SE | P-value |
| --- | --- | --- | --- |
| cg00666422 | -0.00043 | 0.00037 | 0.25 |
| cg03084214 | -0.00015 | 0.00020 | 0.47 |
| cg12083122 | -0.00003 | 0.00012 | 0.78 |
| cg14734794 | -0.00006 | 0.00011 | 0.60 |
| cg16683741 | -0.00007 | 0.00012 | 0.56 |
| cg18603538 | -0.00013 | 0.00023 | 0.58 |
| cg23753947 | 0.00004 | 0.00011 | 0.74 |
| cg24862443 | 0.00009 | 0.00010 | 0.40 |
| cg25435800 | -0.00005 | 0.00018 | 0.79 |
| cg25730670 | 0.00012 | 0.00010 | 0.23 |
| cg07464571 | -0.00020 | 0.00023 | 0.38 |
| cg12782180 | -0.00032 | 0.00035 | 0.36 |
| cg13381984 | -0.00003 | 0.00033 | 0.92 |
| cg19594666 | -0.00027 | 0.00040 | 0.50 |
| cg26814075 | -0.00007 | 0.00029 | 0.81 |
| cg00840332 | -0.00024 | 0.00033 | 0.47 |

Columns: Probe name *= LEP* CpG sites from Illumina Infinium HumanMethylation450 Beadchip; Beta = coefficient from regression model; SE = standard error of coefficient.

**Table S5:** Association between total breastfeeding duration and *LEP* DNAm (23 CpGs) at 10 years.

| Probe name | Beta | SE | P-value |
| --- | --- | --- | --- |
| cg25435800 | 0.00012 | 0.00028 | 0.66 |
| cg11005360 | -0.00024 | 0.00011 | 0.03 |
| cg18603538 | 0.00033 | 0.00018 | 0.07 |
| cg05136031 | -0.00015 | 0.00018 | 0.43 |
| cg15792829 | 0.00017 | 0.00012 | 0.16 |
| cg08349670 | 0.00006 | 0.00011 | 0.56 |
| cg04833007 | -0.00016 | 0.00016 | 0.32 |
| cg00666422 | 0.00005 | 0.00032 | 0.87 |
| cg24862443 | -0.00016 | 0.00012 | 0.18 |
| cg20564991 | 0.00018 | 0.00014 | 0.22 |
| cg23381058 | -0.00049 | 0.00020 | 0.01 |
| cg25730670 | -0.00011 | 0.00011 | 0.32 |
| cg16683741 | 0.00000 | 0.00012 | 0.98 |
| cg23753947 | 0.00017 | 0.00008 | 0.04 |
| cg03084214 | 0.00052 | 0.00023 | 0.03 |
| cg05091920 | 0.00020 | 0.00010 | 0.05 |
| cg12083122 | -0.00013 | 0.00012 | 0.30 |
| cg13381984 | -0.00018 | 0.00031 | 0.57 |
| cg26814075 | -0.00031 | 0.00028 | 0.27 |
| cg19594666 | -0.00010 | 0.00027 | 0.72 |
| cg12782180 | -0.00038 | 0.00030 | 0.20 |
| cg00840332 | -0.00028 | 0.00025 | 0.26 |
| cg11045943 | -0.00047 | 0.00031 | 0.13 |

Columns: Probe name *= LEP* CpG sites from Illumina Infinium MethylationEPIC Beadchip; Beta=coefficient from regression model; SE = standard error of coefficient.

**Table S6:** Association between exclusive breastfeeding duration and *LEP* DNAm (23 CpGs) at 10 years.

| Probe name | Beta | SE | P-value |
| --- | --- | --- | --- |
| cg25435800 | 0.00089 | 0.00056 | 0.11 |
| cg11005360 | -0.00023 | 0.00022 | 0.30 |
| cg18603538 | 0.00081 | 0.00037 | 0.03 |
| cg05136031 | -0.00004 | 0.00037 | 0.90 |
| cg15792829 | 0.00059 | 0.00024 | 0.01 |
| cg08349670 | 0.00000 | 0.00022 | 0.99 |
| cg04833007 | -0.00021 | 0.00032 | 0.51 |
| cg00666422 | -0.00037 | 0.00065 | 0.57 |
| cg24862443 | -0.00030 | 0.00023 | 0.20 |
| cg20564991 | 0.00017 | 0.00029 | 0.56 |
| cg23381058 | -0.00082 | 0.00040 | 0.04 |
| cg25730670 | -0.00019 | 0.00022 | 0.39 |
| cg16683741 | 0.00006 | 0.00024 | 0.81 |
| cg23753947 | 0.00024 | 0.00017 | 0.15 |
| cg03084214 | 0.00069 | 0.00048 | 0.15 |
| cg05091920 | 0.00042 | 0.00021 | 0.05 |
| cg12083122 | -0.00021 | 0.00025 | 0.40 |
| cg13381984 | -0.00015 | 0.00063 | 0.81 |
| cg26814075 | -0.00054 | 0.00057 | 0.34 |
| cg19594666 | 0.00006 | 0.00055 | 0.92 |
| cg12782180 | -0.00039 | 0.00059 | 0.51 |
| cg00840332 | -0.00015 | 0.00050 | 0.77 |
| cg11045943 | -0.00032 | 0.00063 | 0.62 |

Columns: Probe name *= LEP* CpG sites from Illumina Infinium MethylationEPIC Beadchip; Beta = coefficient from regression model; SE = standard error of coefficient.

**Table S7:** Association between BMI trajectories and *LEP* DNAm (at 10 years) at CpGs previously associated with total or exclusive breastfeeding duration (associated P-values have been shown).

**Total breastfeeding**

Trajectory 2

| Probe site | Beta | Standard Error | P-value |
| --- | --- | --- | --- |
| cg03084214 | 1.585 | 5.932 | 0.334 |
| cg11005360 | -23.531 | 12.808 | 0.264 |
| cg23381058 | -1.785 | 3.595 | 0.871 |
| cg23753947 | -3.033 | 15.739 | 0.79 |

Trajectory 3

| Probe site | Beta | Standard Error | P-value |
| --- | --- | --- | --- |
| cg03084214 | 6.585 | 7.330 | 0.349 |
| cg11005360 | 2.986 | 15.504 | 0.366 |
| cg23381058 | 3.183 | 4.277 | 0.034* |
| cg23753947 | -23.567 | 17.710 | 0.946 |

Trajectory 4

| Probe site | Beta | Standard Error | P-value |
| --- | --- | --- | --- |
| cg03084214 | -9.823 | 10.289 | 0.809 |
| cg11005360 | 63.354 | 33.182 | 0.106 |
| cg23381058 | -9.173 | 7.096 | 0.222 |
| cg23753947 | -26.093 | 30.603 | 0.913 |

**Exclusive breastfeeding**

Trajectory 2

| Probe site | Beta | Standard Error | P-value |
| --- | --- | --- | --- |
| cg05091920 | -18.20 | 9.879 | 0.028* |
| cg15792829 | -6.398 | 8.752 | 0.278 |
| cg18603538 | 1.074 | 5.851 | 0.762 |
| cg23381058 | 1.137 | 2.926 | 0.871 |

Trajectory 3

| Probe site | Beta | Standard Error | P-value |
| --- | --- | --- | --- |
| cg05091920 | -15.843 | 10.651 | 0.125 |
| cg15792829 | -17.064 | 9.602 | 0.155 |
| cg18603538 | -1.101 | 6.033 | 0.292 |
| cg23381058 | 4.805 | 3.187 | 0.034* |

Trajectory 4

| Probe site | Beta | Standard Error | P-value |
| --- | --- | --- | --- |
| cg05091920 | -2.662 | 19.796 | 0.694 |
| cg15792829 | 13.102 | 17.240 | 0.507 |
| cg18603538 | 3.976 | 12.232 | 0.669 |
| cg23381058 | -4.90 | 5.857 | 0.222 |

Trajectory 2 = Early persistent obesity; Trajectory 3 = Early transient overweight; Trajectory 4 = delayed overweight. DNA methylation analysed against BMI trajectories with BMI trajectory 1 (normal) as a control. Columns: Probe site = *LEP* CpG sites from Illumina Infinium MethylationEPIC Beadchip, which are significantly associated with total or exclusive breastfeeding; Beta = a one-unit increase in the methylation level for specific CpG is associated with the decrease in the log odds of being in BMI trajectory; Standard Error = standard error of coefficient. P-value < 0.05 was considered significant and indicated by *.

| **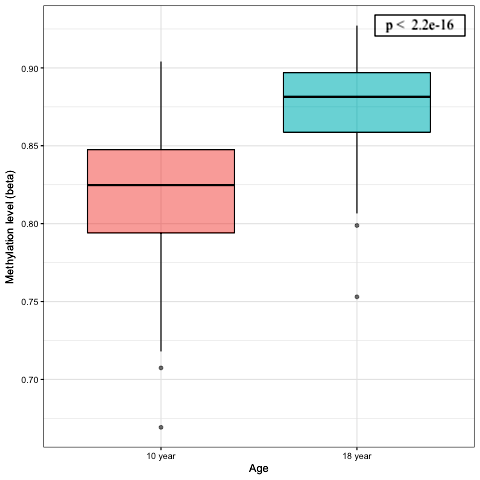** | **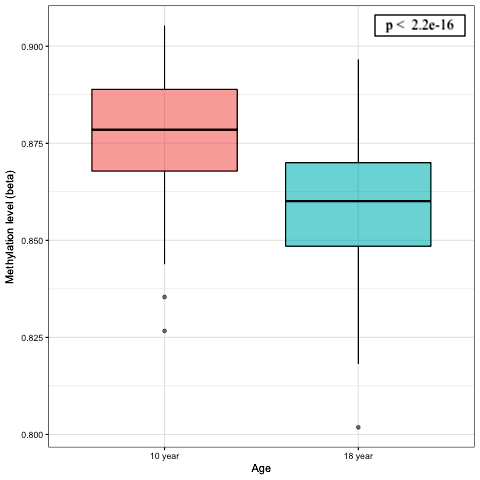** |
| --- | --- |
| a. Methylation level of significant CpG site (cg03084214) in total breastfeeding duration | b. Methylation level of significant CpG site (cg23753947) in total breastfeeding duration |
| **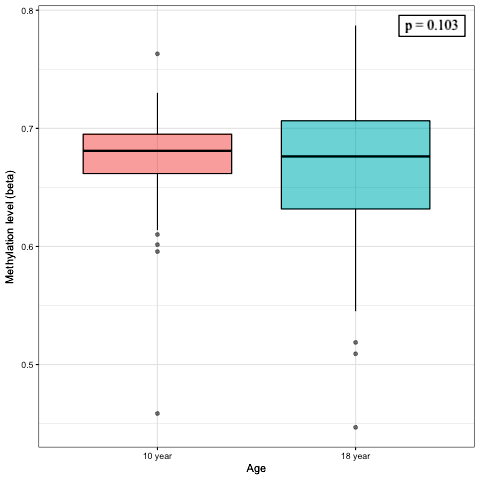** | |
| c. Methylation level of significant CpG site  (cg18603538) in exclusive breastfeeding duration | |

**Figure S1:** Comparisons of methylation level (beta) of significant common CpGs in matched 162 samples (10 and 8 years).

| 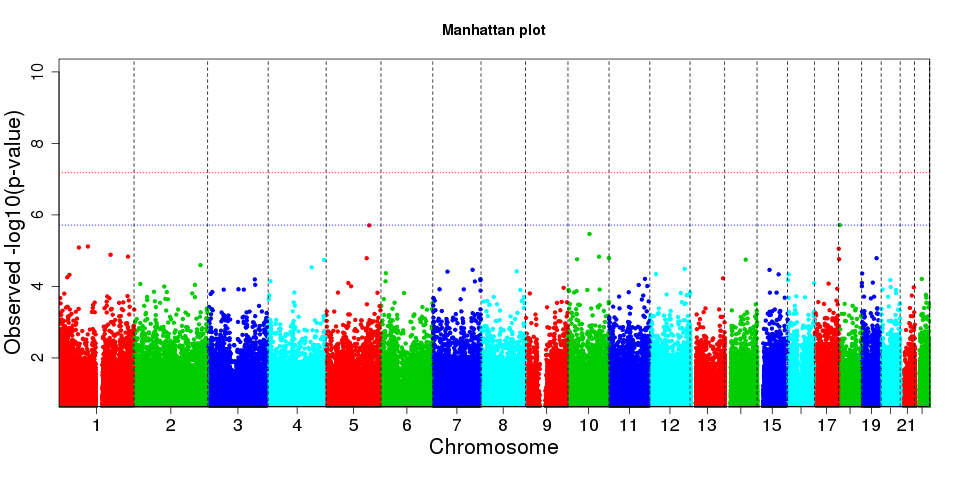 |
| --- |
| 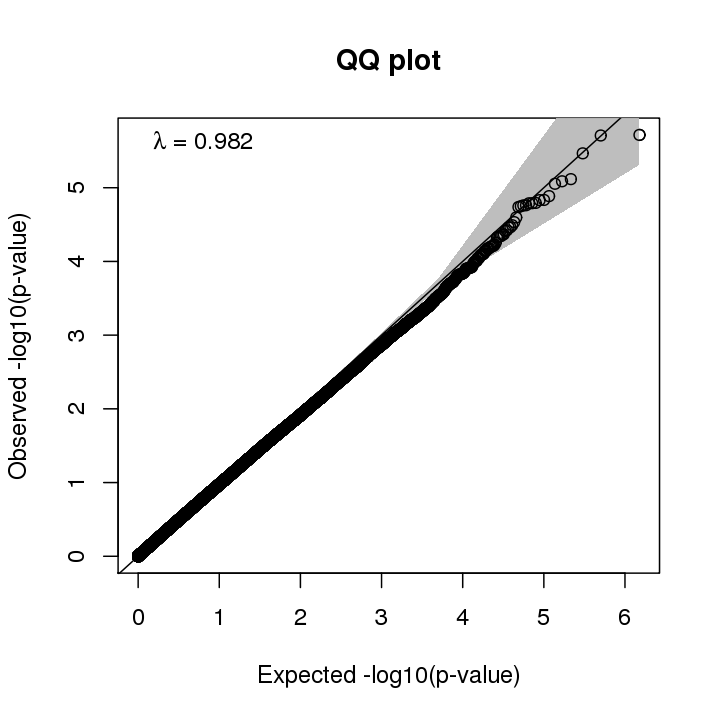 |

**Figure S2:** The Manhattan plot (top) and QQ-plot (bottom) shows the epigenome wide association study results in 10 year old samples. Here DNA methylation has been used as outcome and exclusive breastfeeding duration as exposure adjusted by confounding factors and cell type proportion. In the Manhattan plot, the blue dotted line represents FDR < 0.05 and red dotted line represents Bonferroni threshold.

| 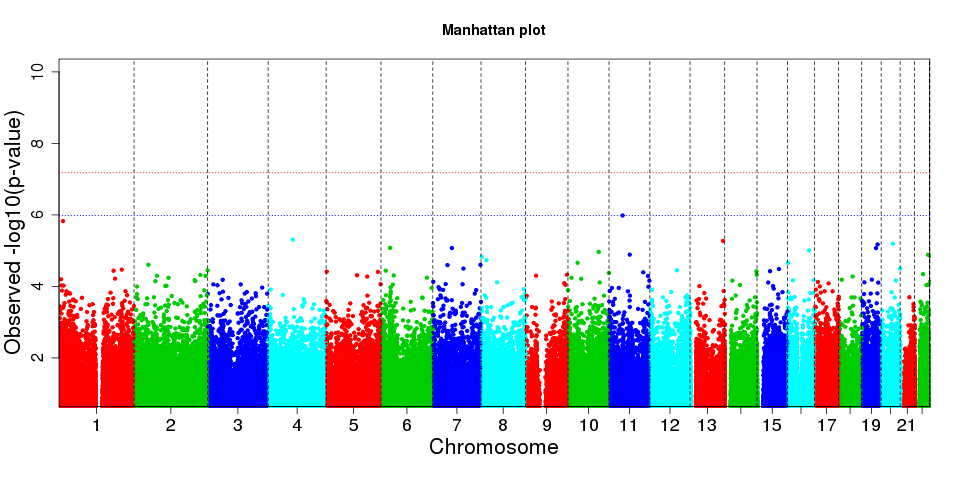 |
| --- |
| 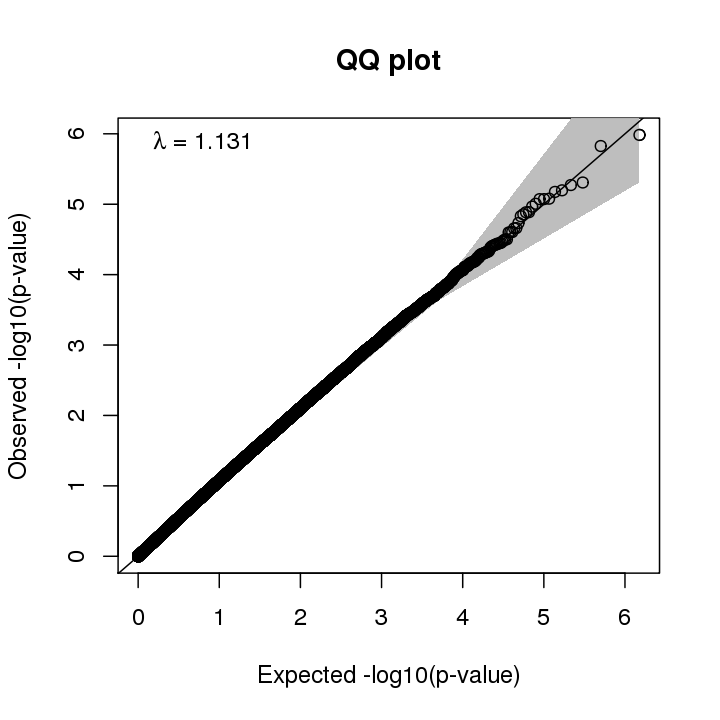 |

**Figure S3:** The Manhattan plot (top) and QQ-plot (bottom) shows the epigenome wide association study results in 10 year old samples. Here DNA methylation has been used as outcome and total breastfeeding duration as exposure adjusted by confounding factors and cell type proportion. In the Manhattan plot, the blue dotted line represents FDR < 0.05 and red dotted line represents Bonferroni threshold.

| 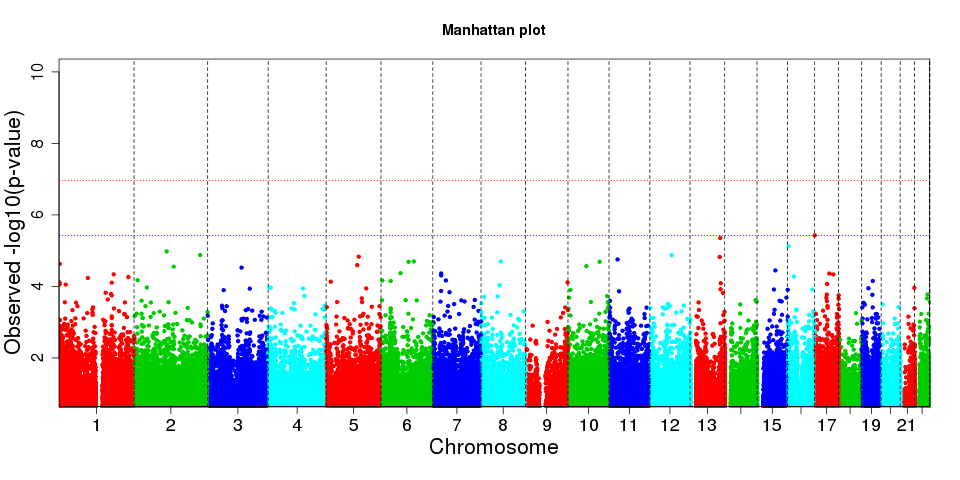 |
| --- |
| 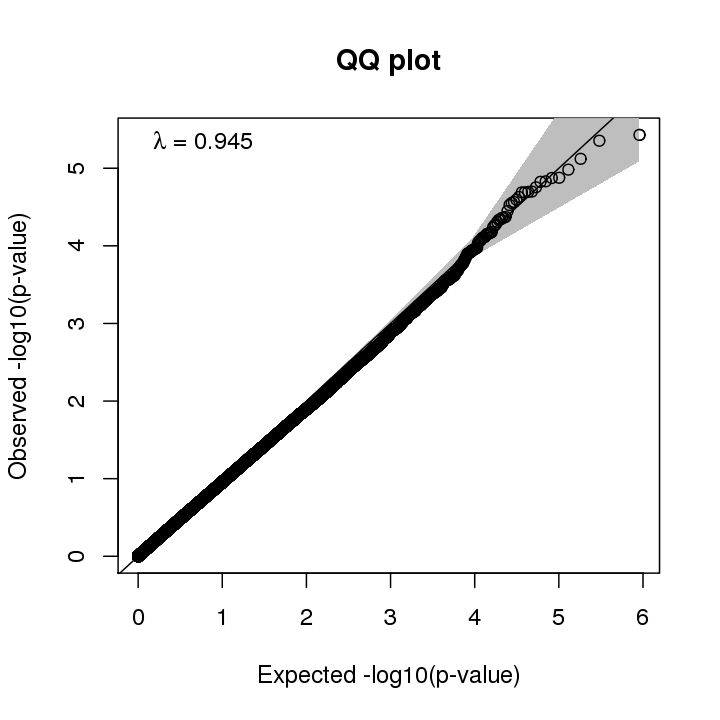 |

**Figure S4:** The Manhattan plot (top) and QQ-plot (bottom) shows the epigenome wide association study results in 18 year old samples. Here DNA methylation has been used as outcome and exclusive breastfeeding duration as exposure adjusted by confounding factors and cell type proportion. In the Manhattan plot, the blue dotted line represents FDR < 0.05 and red dotted line represents Bonferroni threshold.

| 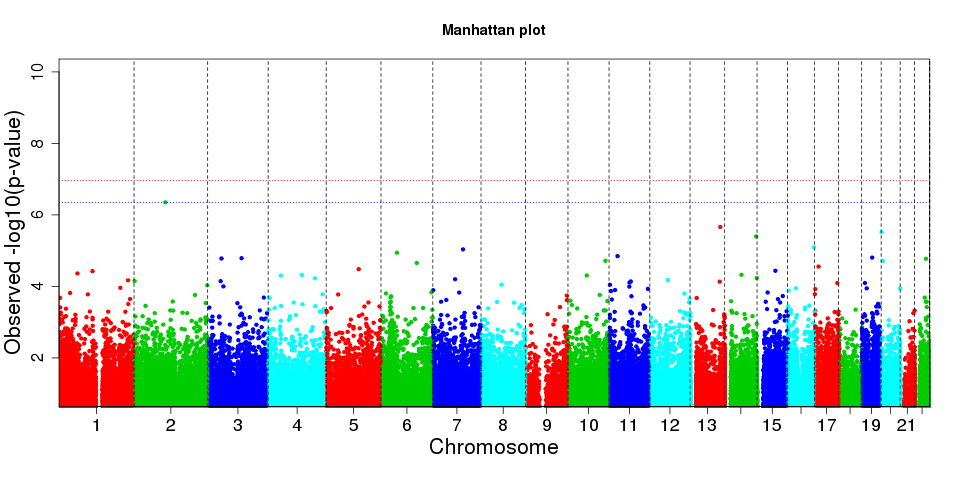 |
| --- |
| 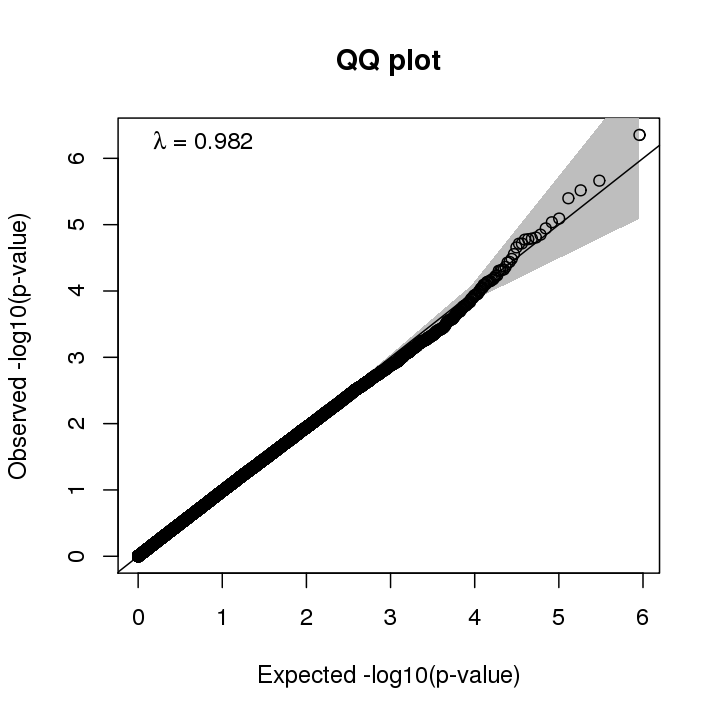 |

**Figure S5:** The Manhattan plot (top) and QQ-plot (bottom) shows the epigenome wide association study results in 18 year old samples. Here DNA methylation has been used as outcome and total breastfeeding duration as exposure adjusted by confounding factors and cell type proportion. In the Manhattan plot, the blue dotted line represents FDR < 0.05 and red dotted line represents Bonferroni threshold.
